# Transplacental transfer of maternal respiratory syncytial virus (RSV) antibody and protection against RSV disease in infants in rural Nepal^☆^

**DOI:** 10.1016/j.jcv.2017.08.017

**Published:** 2017-10

**Authors:** Helen Y. Chu, James Tielsch, Joanne Katz, Amalia S. Magaret, Subarna Khatry, Stephen C. LeClerq, Laxman Shrestha, Jane Kuypers, Mark C. Steinhoff, Janet A. Englund

**Affiliations:** a Department of Medicine, University of Washington, Seattle, WA, USA; b Department of Global Health, George Washington University Milliken School of Public Health, Washington, DC, USA; c Department of International Health, Johns Hopkins Bloomberg School of Public Health, Baltimore, MD, USA; d Department of Laboratory Medicine, University of Washington, Seattle, WA, USA; e Nepal Nutrition Intervention Project-Sarlahi, Kathmandu, Nepal; f Institute of Medicine, Tribhuvan University Teaching Hospital, Kathmandu, Nepal; g Department of Global Health, Cincinnati Children's Hospital Medical Center, Cincinnati, OH, USA; h Center for Clinical and Translational Research, Seattle Children’s Research Institute, Seattle, WA, USA

**Keywords:** Respiratory syncytial virus, Transplacental antibody transfer, Vaccine, Preterm birth

## Abstract

**Background:**

Respiratory syncytial virus (RSV) is the most important viral cause of pneumonia in children. RSV-specific antibody (ab) protects infants from disease, and may be increased by a potential strategy of maternal RSV vaccination.

**Objectives:**

To describe the effect of RSV antibody on RSV infection risk in infants in a resource-limited setting.

**Study design:**

In a prospective study in Nepal, women were enrolled during pregnancy and maternal and infant cord blood were collected at birth. Weekly surveillance for respiratory illness was performed from birth to 180 days. Nasal swabs were tested for RSV by PCR and serum was tested using an RSV antibody microneutralization assay. Antibody concentrations at time of RSV infection were estimated based on a decay rate of 0.026 log_2_/day.

**Results:**

Cord:maternal RSV antibody transfer ratio was 1.03 (0.88–1.19), with RSV antibody concentration of log_2_ 11.3 and log_2_ 11.7 in 310 paired maternal and infant samples, respectively. Cord blood RSV antibody was log_2_ 12.1 versus 11.6 in those with or without RSV infection (P = 0.86). Among infants with RSV infection, estimated RSV antibody concentration at time of infection did not differ in infants with upper (n = 8; log_2_ 10.7) versus lower respiratory tract infection (n = 21; log_2_ 9.8; P = 0.37). Cord blood RSV antibody concentrations did not correlate with age at primary RSV infection (R = 0.11; P = 0.57).

**Conclusions:**

Transplacental transfer of RSV antibody from mother to the fetus was highly efficient in mother-infant pairs in rural Nepal, though higher antibody concentrations were not protective against earlier or more severe RSV infection in infants.

## Highlights

• Transplacental transfer of RSV antibody was highly efficient in Nepal.
• Cord blood RSV antibody concentrations were not associated with age at RSV illness.
• Estimated RSV antibody concentrations did not correlate with disease severity.

## 1 Background

Pneumonia is the leading cause of childhood deaths in developing countries. Respiratory syncytial virus (RSV) is the most important cause of viral pneumonia in infants worldwide, with an estimated global burden of 64 million infections and 160,000 deaths annually. There are limited pharmacologic therapies and no licensed vaccine against RSV [1]. Advances in immunology and virology have accelerated the RSV vaccine field significantly in the past decade, and the World Health Organization has estimated that an RSV vaccine will be in clinical use within the next 5–10 years [2]. However, infants at high risk of mortality from RSV also have relatively immature immune systems that might preclude protection from even the most effective vaccine candidate [3]. Maternal RSV antibody is protective against severe disease in neonates, and palivizumab, a high-titered RSV-specific monoclonal antibody, is administered to high-risk infants in developed countries to prevent hospitalization [4], [5]. However, 99% of deaths due to RSV occur in resource-limited settings, making vaccine development critical in preventing RSV-associated mortality [6].

Maternal immunization against RSV has the potential to protect the mother from illness, the fetus from adverse birth outcomes, and the infant from early disease through transplacental antibody transfer. Maternal vaccines for influenza, tetanus, and pertussis are safe, immunogenic, and efficacious for both mother and infant [3]. RSV transplacental antibody transfer is efficient and antibody half-life is approximately one month [7]. Higher antibody levels in the mother may extend the period of protection from RSV disease in the infant, and thereby reduce infant morbidity and mortality due to RSV in early infancy. The majority of antibody transfer occurs during the third trimester of pregnancy, and is impacted by many variables including maternal antibody titers, gestational age at birth, total immunoglobulin G (IgG) concentrations, and maternal infection with HIV or malaria [3]. Prospective epidemiologic studies in the United States have shown that higher RSV antibody concentrations are associated with less severe disease in infants [5], [8]. However, the greatest burden of RSV is in developing countries with high rates of low birth weight and preterm births [9].

## 2 Objectives

We seek to evaluate the factors that impact RSV transplacental antibody transfer and the relationship between cord blood RSV antibody concentrations and incidence, severity, and timing of RSV infection in young infants in a developing country setting.

## 3 Study design

We conducted an analysis using samples and clinical data collected in a community-based, prospective placebo-controlled, randomized trial of maternal influenza immunization of 3693 pregnant women and their infants conducted in rural southern Nepal from 2011 to 2014 [10], [11]. Briefly, respiratory illness episodes were identified through longitudinal household-based active weekly surveillance of infants from birth until 180 days. Respiratory illness was defined as the presence of any of the following: fever, cough, difficult or rapid breathing, rhinorrhea, wheeze, or otorrhea. Infants with a respiratory illness had a mid-nasal swab taken. From March 2012 to October 2013, paired maternal and cord blood samples were collected at the time of delivery in a convenience sample of 310 mother-infant pairs.

Mid-nasal swabs were tested for RSV and 11 additional respiratory viruses using a real-time PCR (qPCR) assay [12]. Serum samples were tested for RSV antibody using a microneutralization assay [13]. Briefly, serum was added in serial two-fold dilutions to a 96-well plate, and incubated with RSV A2 virus for 30 min prior to addition of Hep2 cells followed by a 3 day incubation at 37**°**C 5% CO2. The assay was developed with a monoclonal antibody against the RSV fusion surface protein, and a secondary horseradish-peroxidase conjugated goat anti-mouse IgG. The endpoint concentration was defined as the 50% cutoff between positive and negative controls. Positive and negative controls, as well as reference sera were included on each run (Appendix A: Reference sera results). Antibody concentrations at time of RSV infection were estimated based on a decay rate of log_2_ 0.026/day [7].

Birth weight was included if measured <72 h after birth. Low birth weight was defined as <2500 g. Preterm birth was defined as <37 weeks gestation. Gestational age was calculated according to the last menstrual period based on a house-to-house census conducted every five weeks of all women of childbearing age. Date of last menstrual period, therefore, was used to estimate gestational age with a five-week recall period. Small-for-gestational-age (SGA) was defined using the Intergrowth-21 criteria [14].

An RSV illness was defined as a respiratory illness with RSV detected by qPCR. Lower respiratory tract infection (LRTI) was defined using the World Health Organization Integrated Management of Childhood Illness criteria as cough and/or difficulty breathing with age-specific tachypnea or wheezing according to an evaluation by a trained field worker [15]. LRTI also included severe or very severe pneumonia, defined as cough and/or difficulty breathing with chest wall indrawing, lethargy or unconsciousness, convulsions, cyanosis or inability to feed/drink or vomiting everything. Upper respiratory tract infection (URTI) was an RSV illness episode that did not meet criteria for LRTI [15].

Data was analyzed using SAS (Cary, NC, USA). Categorical measures were compared by chi-squared tests and continuous variables by two-sample *t*-tests. For the evaluation of cord:maternal antibody ratio, samples from mother-infant pairs where either sample had undetectable titers were excluded from the analysis (n = 2 maternal samples; n = 2 cord samples). Examination of the impact of covariates on antibody ratio between infant and mother also used linear regression with ratio as the outcome. The influence of the antibody ratio on RSV infection incidence was examined using Poisson regression with RSV as the outcome, log follow-up time as the exposure, and antibody ratio as the only predictor.

IRB approval for the study was obtained from the Johns Hopkins University Bloomberg School of Public Health, Seattle Children's Hospital, Cincinnati Children's Hospital, the Institute of Medicine at Tribhuvan University, and the Nepal Health Research Council. The trial in which this sub-study was conducted is registered at Clinicaltrials.gov (NCT01034254).

## 4 Results

From March 2012 to October 2013, paired maternal and cord blood samples at the time of delivery were collected from a subset of 310 (9%) of 3646 mother-infant pairs in the parent study. As compared to the overall study population, these pairs were more likely to have a breastfed infant (85% vs. 78%; P = 0.0026), and less likely to be preterm (9% vs. 13%; P = 0.019) or low birth weight (17% vs. 26%; P = 0.001; Table 1). No difference in RSV incidence was observed between the infants who were or were not included in this substudy (224/1000 vs. 212/1000 person-years; P = 0.86).

**Table 1:** Comparison of baseline characteristics in mother-infant pairs included or excluded in this substudy of RSV transplacental antibody transfer.

	Median (range) or number (%)		
	Included (n = 310)	Excluded (n = 3383)	p-value
Mother’s characteristics			
Maternal age	23 (15–39)	23 (13–45)	0.37
Number of years of education	5 (0–16)	5 (0–18)	0.19
Any previous children	183 (59%)	1955 (58%)	0.65
No. of children <15 yrs age	1 (0–10)	2 (0–13)	0.28
Breastfeeding	265 (85%)	2637 (78%)	0.0026
Smoker in household	118 (40%)	1392 (44%)	0.18

Infant characteristics			
Male sex of infant	161 (52%)	1776 (53%)	0.85
Preterm birth (<37 weeks)	26 (9%)	444 (13%)	0.019
Low birth weight	51/305 (17%)	628/2436 (26%)	0.001
Infant birth weight, kg	2.85 (1.51–3.97)	2.79 (0.82–4.80)	0.010
Small for gestational age	154 (50%)	1342 (55%)	0.13
RSV infection (ever)	30 (9%)	281 (9%)	0.60
RSV infection incidence	30 per 134 p-y = 224/1000 p-y	281 per 1327 p-y = 212/1000 p-y	0.86

**Fig. 1:**
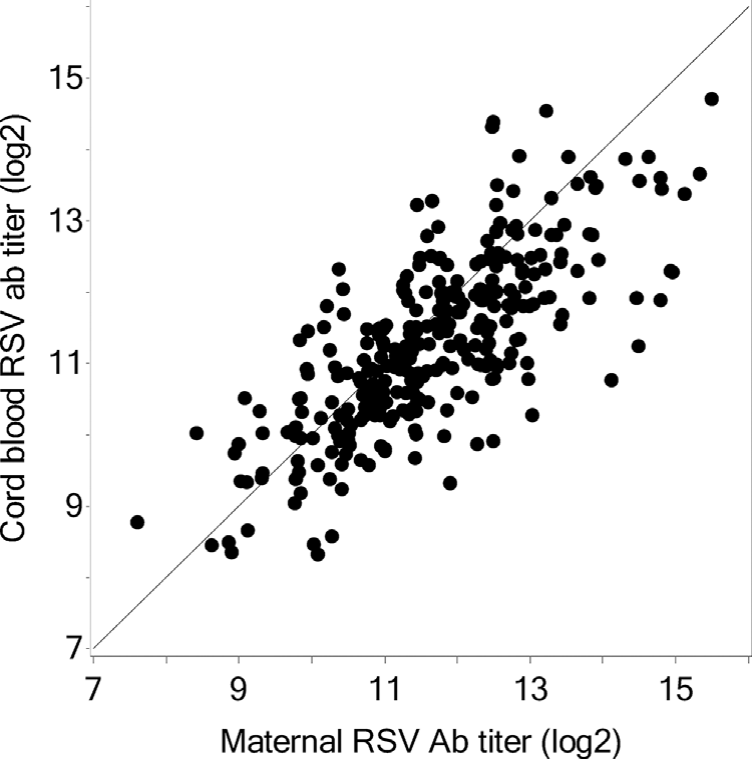
Comparison of RSV antibody in maternal (x-axis) and infant cord blood (y-axis) at time of delivery in 310 mother-infant pairs (Pearson’s correlation coefficient 0.77, p < 0.0001).

Mean RSV log_2_ antibody concentration was 11.3 [SD: 1.2] and 11.7 [SD: 1.3] in the maternal and infant cord blood, respectively (Table 2). Two maternal and two infant cord blood samples had undetectable antibody titers, and these four pairs were excluded from analysis. The geometric means of the original antibody concentrations for maternal and infant cord blood are 1760 and 2020, respectively. The RSV cord:maternal antibody transfer ratio was 1.03 (95% CI: 0.88, 1.19). Maternal and infant RSV antibody concentrations were highly correlated (R = 0.77; P < 0.0001; Fig. 1).

**Table 2:** Maternal and Infant cord RSV antibody concentrations and transfer ratio.

	Mother		Infant		Cord:maternal antibody transfer ratio (95% CI)
	Mean log_2_ Ab (SD)	GMT	Mean log_2_ Ab (SD)	GMT	
Overall	11.3 (1.2)	2480	11.7 (1.3)	3260	1.03 (0.89, 1.19)
RSV-positive	11.4 (1.4)	2710	12.1 (1.3)	4460	1.07 (0.89, 1.26)
RSV-negative	11.3 (1.2)	2450	11.6 (1.3)	3160	1.03 (0.89, 1.18)
Preterm birth (<37 weeks)	11.5 (1.3)	2990	12.0 (1.3)	3970	1.04 (0.88, 1.20)
Full term birth	11.3 (1.2)	2450	11.6 (1.3)	3200	1.04 (0.89, 1.19)
Small for gestational age	11.2 (1.2)	2430	11.7 (1.3)	3280	1.04 (0.89, 1.19)
Appropriate for gestational age	11.3 (1.3)	2560	11.7 (1.2)	3240	1.03 (0.88, 1.19)

**Fig. 2:**
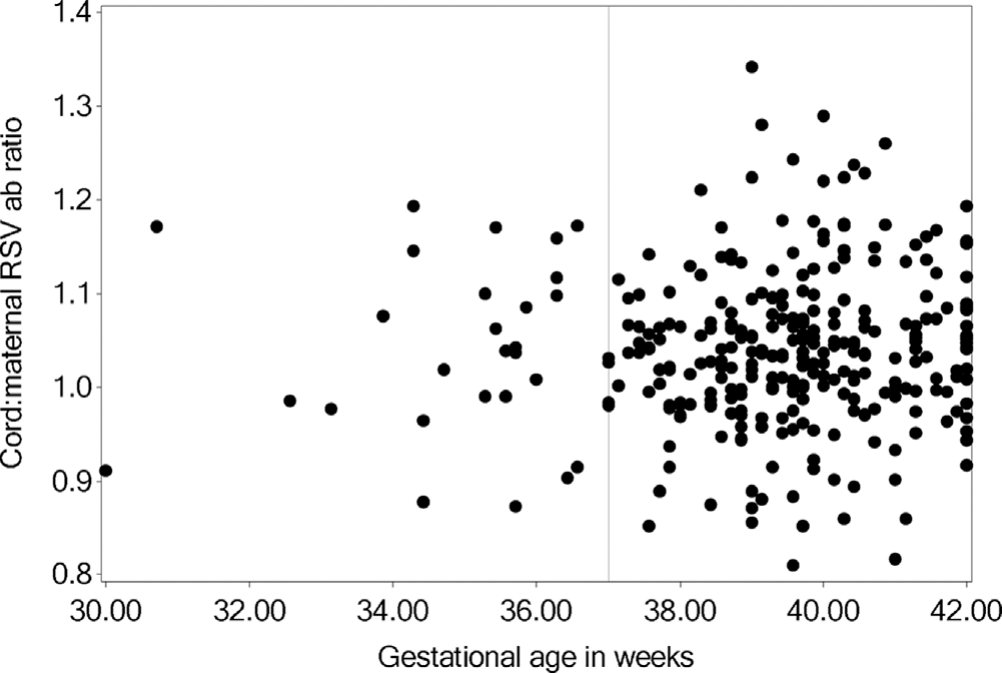
Comparison of cord:maternal RSV antibody transfer ratio by gestational age at delivery. No significant correlation was found between RSV antibody ratio and gestational age in weeks at birth (R = 0.05; P = 0.37).

**Fig. 3:**
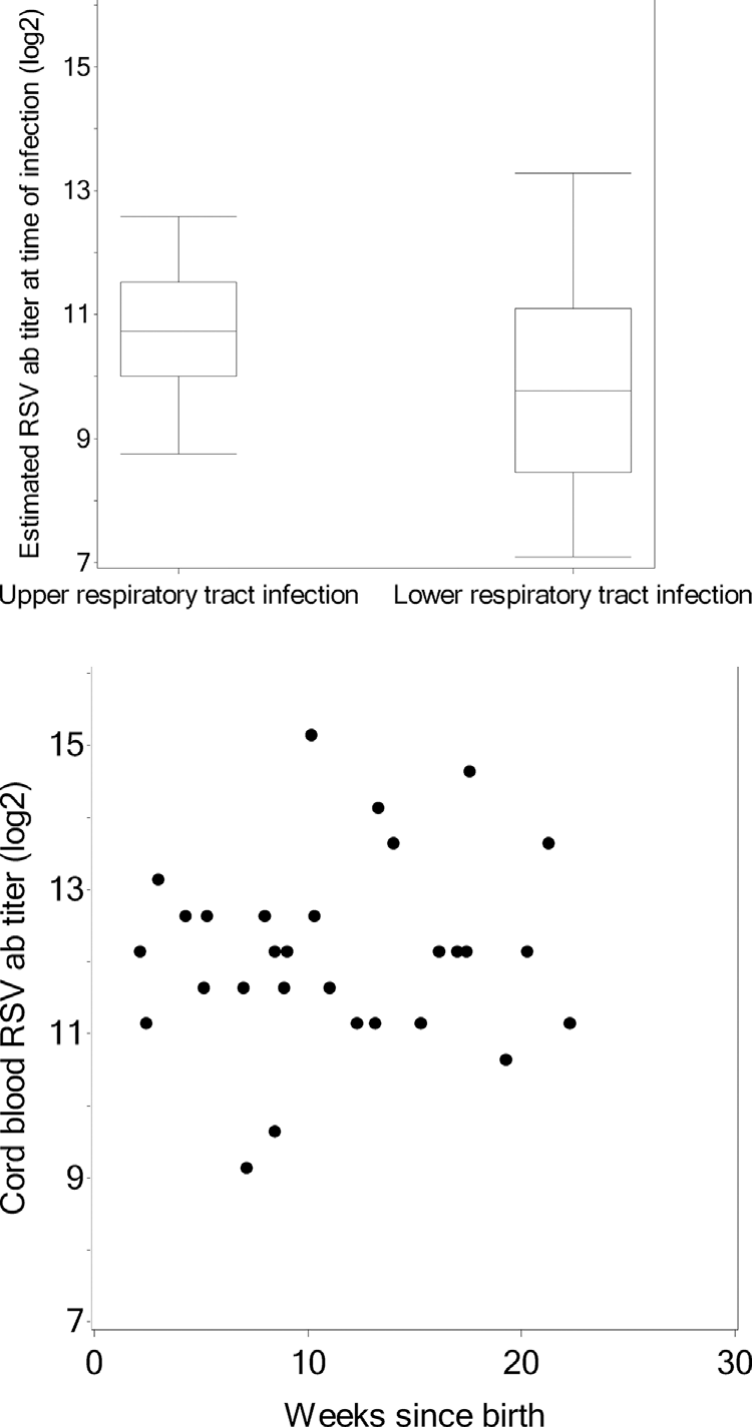
3A. Side-by-side boxplot comparing estimated RSV antibody concentrations at time of infection in infants with RSV upper respiratory tract versus lower respiratory tract infection. Infant antibody concentrations at time of RSV infection were estimated based on a decay rate of 0.026 log_2_ concentration/day. Of the 30 infants with RSV infection, 21 had LRTI. Estimated RSV antibody concentrations at the time of infection did not differ significantly between infants with URTI vs. LRTI (10.7 [1.2] vs. 9.8 [1.7], respectively; P = 0.37). 3B. Scatterplot comparing weeks at RSV illness against cord blood RSV antibody concentrations shows no relationship between age at illness and cord blood antibody titers among infants with RSV infection (R = 0.11; P = 0.57; Fig. 3B).

Altogether, 30 (9%) infants in the original 310 mother-infant pairs had a subsequent symptomatic RSV infection. Maternal log_2_ RSV antibody was 11.4 in infants with subsequent RSV infection, and 11.3 in those without RSV infection (P = 0.30; Table 2). Cord blood RSV antibody was 12.1 in infants with and 11.6 in those without RSV infection (P = 0.86). Maternal RSV antibody was 11.5 in infants born preterm, and 11.2 born full-term (P = 0.79). Surprisingly, antibody transfer ratio was higher in those with versus without RSV infection (1.06 (n = 30) vs. 1.03 (n = 280), respectively; P = 0.025; Table 3). Because transplacental antibody transfer has been shown to be impacted by gestational age at birth, we evaluated the frequency of preterm birth in infants with and without RSV infection. The proportion of those with preterm birth in infants with RSV infection was 10% (n = 30), and 9% in those without RSV infection (n = 280). Transfer ratio was not impacted by maternal age, maternal education, maternal parity, breastfeeding, or smoking in the household. Antibody transfer ratio was significantly lower if the infant was male (1.02 vs. 1.05; P = 0.0017), but did not differ with preterm, low birthweight, or small for gestational age births. Increasing maternal parity was significantly associated with increased transplacental antibody transfer ratio. The influence of antibody ratio on RSV infection incidence was examined using Poisson regression with RSV as the outcome, log follow-up time as the exposure, and antibody ratio as the only predictor. In that model the incidence of RSV was not found to differ by antibody ratio (p = 0.18). No significant correlation was found between RSV antibody ratio and gestational age in weeks at birth (R = 0.05; P = 0.37; Fig. 2). Of 30 infants with RSV infection, 21 had LRTI. Estimated RSV antibody concentrations at the time of infection did not differ significantly between infants with URTI vs. LRTI (10.7 [1.2] vs. 9.8 [1.7], respectively; P = 0.37: Fig. 3A). Additionally, cord blood RSV antibody concentrations did not correlate with age at primary RSV infection (R = 0.11; P = 0.57; Fig. 3B)

**Table 3:** RSV cord:maternal antibody transfer ratio by baseline covariates utilizing univariable regression.

Covariate	Frequency	RSV cord:mother antibody ratio if yes	RSV cord:mother antibody ratio if no	p-value
Maternal characteristics				
Mother above median age of 23 years	130 (45%)	1.03	1.04	0.44
Mother above median education of 5 yrs	139 (48%)	1.03	1.03	0.69
Any previous children	183 (59%)	1.02	1.04	0.012
Breastfeeding	265 (85%)	1.03	1.03	0.71
Smoking by household members	118 (40%)	1.03	1.03	0.85

Infant characteristics				
Male sex	161 (52%)	1.02	1.05	0.0017
Preterm birth (birth <37 wks)	26 (9%)	1.04	1.04	0.92
Low birth weight (weight < 2500 g)	51/305 (17%)	1.04	1.03	0.75
Infant above median birthweight of 2800 g	167/305 (55%)	1.03	1.04	0.62
Small for gestational age	154 (50%)	1.04	1.03	0.50
RSV infection, ever	30 (9%)	1.07	1.03	0.025

## 5 Discussion

In a prospective longitudinal study conducted in rural Nepal, RSV serum antibody transfer from mother to infant was highly efficient. However, no relationship was found between RSV antibody concentrations and RSV infection risk, severity or timing of infection in infants.

There is no vaccine for RSV. However, maternal RSV vaccines are currently in clinical trials with the goal to vaccinate pregnant women to increase antibody concentration in infants at birth. Previous studies have shown that disease-specific antibody transfer occurs primarily in the third trimester and antibody concentrations in the infant often exceed that of the mother by time of birth [16]. In this study, the majority of infants had higher antibody concentrations at birth than their mother, confirming efficient RSV antibody transfer in South Asia. These results are similar to our previous findings in a more affluent, urban population in Dhaka, Bangladesh [7]. We did not find a decreased RSV antibody ratio in infants born preterm. This is in line with recent studies of maternal pertussis vaccination that have found that second trimester vaccination may be associated with increased cord blood antibody titers at birth [17]. Together these data demonstrate that timing of maternal vaccines should potentially include the second trimester to maximize the ability to vaccinate women at risk for preterm birth. This may be particularly relevant in settings where 15% of the infants are preterm, a known risk factor for severe RSV disease [18], [19].

This study was conducted in rural southern Nepal, a setting with limited antenatal care and frequent home-based deliveries. The strengths of this study included collection of maternal and infant cord blood at home births, use of weekly active home-based surveillance for respiratory illness in infants from birth to six months of age, and the ability to stage severity of RSV disease by trained health care workers at home visits. This allowed for characterization of the effect of RSV antibody transfer ratio on risk of primary RSV infection in a developing country setting broadly representative of South Asia. Surprisingly, no effect of RSV antibody was found on incidence, severity, or timing of RSV infection in infants in this study. Among the RSV-infected infants, those with severe disease had lower estimated serum antibody concentrations at the time of infection, though the numbers were small and this was not significant.

In observational U.S. studies, higher cord blood RSV antibody concentrations were associated with older age at infection, decreased risk of infection and hospitalization, and less severe disease among those hospitalized [5], [8], [20], [21]. Palivizumab, a monoclonal antibody against RSV, has also shown to be effective in prevention of RSV hospitalization in high-risk infants [22]. However, studies in Kenya and Alaska have also not shown an effect of cord blood antibody on RSV infection risk [23], [24]. Alaska-Native infants have the highest rates of RSV hospitalization in the world [25]. Many risk factors in the Alaska-Native population are shared with this study population, including household crowding and lack of running water. In the Alaska-Native study, no difference was found in cord blood antibody concentrations in infants hospitalized with RSV as compared to community controls. They did find lower antibody concentrations in infants with severe disease, though sample sizes were small and this did not reach significance [26]. However, a follow-up study in the same population showed that prophylaxis with palivizumab reduced RSV hospitalization rates, suggesting that higher antibody concentrations than those achieved by natural infection are protective. In the Kenya study, Nyiro and colleagues utilized a birth cohort of infants intensively surveyed for RSV. Like Singleton et al., they found no association between higher cord blood RSV antibody and disease risk in infants hospitalized for RSV and age-matched controls. Our study differed from both the Alaska and the Kenya studies in that we utilized prospective community-based surveillance for respiratory illness, allowing us to capture infants not seen by a physician or at a hospital. Hospitalization may not be an adequate correlate of disease severity in developing countries, given the many barriers that limit access to health care. In prior studies in this population, we found no association between RSV disease severity and health care-seeking [19]. However, utilizing home-based staging for disease severity, we found similar results to both Alaska and Kenya.

There are several potential explanations for these findings. The first is that in settings where infants are exposed to a high viral inoculum from repeated exposures to other young children, serum antibody generated by natural infection in mothers is insufficient to protect very young infants. The second is that mucosal antibody, such as nasal RSV IgA, may be protective from infection, as shown in experimental adult human infection models [27], while serum antibody may protect from severe lower respiratory tract disease. In elderly adults, both mucosal and serum antibody are protective, though the levels of each are not correlated [28]. We found a lower serum antibody concentration in infants with RSV LRTI as compared to those with RSV URTI, similar to that found by Singleton et al., though this effect was not significant. Finally, to our knowledge, the role of serum RSV neutralizing antibody on protection from virologically confirmed RSV infection has not been well-characterized in community-based developing country settings. These differ significantly from developed countries, where birthweights are higher, households are less crowded, and malnutrition is uncommon. It may be that protection from RSV infection in rural Nepal is multifactorial, and establishing a correlate of protection will require a combination of multiple endpoints, rather than serum neutralizing antibody concentrations alone.

We additionally found that increasing maternal parity and female sex of the infant were both significantly associated with increased transplacental antibody transfer ratio. To our knowledge, no other studies have found a relationship between increasing maternal parity and greater transplacental antibody transfer. It is possible that parity is a marker for increased exposure to other children with RSV, though in previous studies in this cohort we did not find that increasing RSV infection risk was seen with increasing parity. Male infants are known to have more severe LRTI than female infants, but it is not been previously shown that gender is associated with decreased antibody transfer. Larger studies to address these issues would be warranted.

Limitations of our study included the small number of infants with RSV infection, limiting our ability to detect a difference in antibody concentrations in infants with URTI and LRTI. A more accurate evaluation of the protective effect of serum antibody would be a comparison of antibody in RSV-uninfected and infected infants matched by birth month; however, our numbers were too small to do this analysis. Additionally, we did not survey for asymptomatic infection, though primary RSV infection is rarely asymptomatic. We also did not measure nasal or breast milk RSV IgA concentrations, which may be additional factors associated with protection. We additionally acknowledge that differences are observed in risk factors for RSV infection in mother-infant pairs for whom serum was available for testing, including a lower incidence of preterm birth than the overall cohort. This is due primarily to the difficulty in obtaining cord blood at time of birth in a region of the world where births occurred at home with limited medical assistance. Preterm birth is a risk factor for RSV incidence in our study. However, no differences were observed in RSV incidence between the two cohorts. It is possible that the preterm infants who were included in this convenience study had additional protective factors that decreased their risk of RSV infection, such as socioeconomic status, and it is possible that these protective factors may influence RSV antibody titers.

In conclusion, we find that RSV transplacental antibody transfer from the pregnant woman to her infant is efficient in a subtropical rural developing countrysetting, but that higher cord blood RSV antibody titers are not protective against earlier or more severe disease in young infants.

## Funding

This work was supported by National Institute of Allergy & Infectious Diseases at the National Institutes of Health [K23-AI103105 to HYC] and the Bill & Melinda Gates Foundation, Seattle, WA [50274 to all authors]. The funders had no role in study design, collection or analysis of data, manuscript preparation, or in decision to submit the publication.

## Competing interests

JAE has received research support from Gilead, Chimerix, GlaxoSmithKline, and Roche, has received payment for lectures from Abbvie, and serves as a consultant for GlaxoSmithKline and Gilead. MCS serves on the board of the Novartis Vaccine Institute for Global Health. ASM consults for AiCuris. All other authors declare no conflicts of interest.

## Ethical approval

IRB approval for the study was obtained from the Johns Hopkins University Bloomberg School of Public Health, Seattle Children's Hospital, Cincinnati Children’s Hospital, the Institute of Medicine at Tribhuvan University, and the Nepal Health Research Council.

## Author contributions

HYC and JAE conceived the study. HYC performed the laboratory testing, initial data analysis and wrote the manuscript. JKa, JT, MS, JKu, SC, LS and SK were responsible for design of the primary trial and collection of the study samples and clinical data for this substudy. ASM performed the statistical analysis. All authors contributed to and approved the manuscript.
