# Supplementary material for: Transplacental transfer of maternal respiratory syncytial virus (RSV) antibody and protection against RSV disease in infants in rural Nepal
Source: J Clin Virol. 2017 Oct;95:90–5. doi: 10.1016/j.jcv.2017.08.017 (PMC5625849; doi:10.1016/j.jcv.2017.08.017)
Supplement: Supplementary file 1 [file mmc1.docx]

**Appendix A**

Standard reference sera included on the RSV antibody microneutralization assay with results as compared to the expected titer range provided by the Biodefense and Emerging Infections Research Resources Repository.

| Reference Sera | **Expected titer range** provided by Biodefense and Emerging Infections Research Resources Repository | Chu Laboratory  Mean log_2_ antibody titer (SD) | Standard Deviation |
| --- | --- | --- | --- |
| BEI NR-4020  Wyeth lot 6594  (501) | 8.79+/-6.48 | 8.95 | 1.14 |
| BEI NR-4021  Wyeth lot 6937 - high  (512) | 10.88+/-9.75 | 10.56 | 0.84 |
| BEI NR-4022  Wyeth lot 6938 - medium  (513) | 7.81+/-5.81 | 8.61 | 1.12 |
| BEI NR-4023 Wyeth lot 6939 - low  (514) | 8.28+/-2.81 | 8.45 | 1.17 |
| BEI NR-21973  CBER reference Ig lot RSV-1 | 7.56+/-5.21 | 11.95* | 0.94 |
| Intravenous immunoglobulin |  | 10.70 | 0.97 |

*We test this reference sample undiluted, reference range results refer to sample tested at 1:100 dilution.
